# Deconvolution of Bulk Gene Expression Profiles with Single-Cell Transcriptomics to Develop a Cell Type Composition-Based Prognostic Model for Acute Myeloid Leukemia

**DOI:** 10.3389/fcell.2021.762260

**Published:** 2021-11-12

**Authors:** Chengguqiu Dai, Mengya Chen, Chaolong Wang, Xingjie Hao

**Affiliations:** Department of Epidemiology and Biostatistics, Key Laboratory for Environment and Health, School of Public Health, Tongji Medical College, Huazhong University of Science and Technology, Wuhan, China

**Keywords:** cell type composition, gene expression profiles, transcriptome deconvolution, prognostic model, acute myeloid leukemia

## Abstract

Acute myeloid leukemia (AML) is one of the malignant hematologic cancers with rapid progress and poor prognosis. Most AML prognostic stratifications focused on genetic abnormalities. However, none of them was established based on the cell type compositions (CTCs) of peripheral blood or bone marrow aspirates from patients at diagnosis. Here we sought to develop a novel prognostic model for AML in adults based on the CTCs. First, we applied the CIBERSORT algorithm to estimate the CTCs for patients from two public datasets (GSE6891 and TCGA-LAML) using a custom gene expression signature reference constructed by an AML single-cell RNA sequencing dataset (GSE116256). Then, a CTC-based prognostic model was established using least absolute shrinkage and selection operator Cox regression, termed CTC score. The constructed prognostic model CTC score comprised 3 cell types, GMP-like, HSC-like, and T. Compared with the low-CTC-score group, the high-CTC-score group showed a 1.57-fold [95% confidence interval (CI), 1.23 to 2.00; *p* = 0.0002] and a 2.32-fold (95% CI, 1.53 to 3.51; *p <* 0.0001) higher overall mortality risk in the training set (GSE6891) and validation set (TCGA-LAML), respectively. When adjusting for age at diagnosis, cytogenetic risk, and karyotype, the CTC score remained statistically significant in both the training set [hazard ratio (HR) = 2.25; 95% CI, 1.20 to 4.24; *p* = 0.0119] and the validation set (HR = 7.97; 95% CI, 2.95 to 21.56; *p <* 0.0001]. We further compared the performance of the CTC score with two gene expression-based prognostic scores: the 17-gene leukemic stem cell score (LSC17 score) and the AML prognostic score (APS). It turned out that the CTC score achieved comparable performance at 1-, 2-, 3-, and 5-years timepoints and provided independent and additional prognostic information different from the LSC17 score and APS. In conclusion, the CTC score could serve as a powerful prognostic marker for AML and has great potential to assist clinicians to formulate individualized treatment plans.

## Introduction

Acute myeloid leukemia (AML) is characterized by malignant clonal hematopoiesis, which is caused by the accumulation of somatic mutations in hematopoietic stem cells (HSCs) or downstream progenitors (Yamashita et al., 2020). Among diverse leukemia subtypes, AML accounts for most leukemia patients and leukemia-related deaths, and the incidence has been continuously increasing in recent years (Ghazawi et al., 2019; Roman et al., 2016; Shallis et al., 2019). The average 5-years overall survival (OS) probability is approximately 24% by 2016 in the United States, the fifth worst by cancer types, and 17% between 2000 and 2007 in Europe (De Angelis et al., 2015; Shallis et al., 2019). Therefore, accurately stratifying the prognosis is of great significance to formulate individualized treatment plans for AML patients.

As high-throughput sequencing technology becomes affordable, the comprehensive landscape of AML driver mutations has been gradually revealed (Cancer Genome Atlas Research et al., 2013; Papaemmanuil et al., 2016). Identifying the genetic abnormalities, including cytogenetic alterations and molecular variants, greatly contributes to the prognostic assessments for AML patients at diagnosis (Grimwade et al., 2010; Marcucci et al., 2011). Nevertheless, existing prognostic stratifications, such as the 2017 European LeukemiaNet (ELN) risk stratification (Dohner et al., 2017), still require further improvement due to the diversity and heterogeneity of the AML-related genetic abnormalities within and across patients. Some studies attempted to seek novel prognostic markers using gene expression profiles (GEPs), such as the 17-gene leukemic stem cell score (LSC17 score) (Ng et al., 2016) and the AML prognostic score (APS) (Docking et al., 2021). Some of these expression-based prognostic markers showed great performances in evaluating prognosis for AML patients. However, it is difficult to interpret how the genes used to compute the prognostic score affect the prognosis.

It has been suggested that the cell type compositions (CTCs) in the tumor microenvironment are associated with tumor growth, progression, invasion, and metastasis (Hanahan and Weinberg., 2011). Recently, with the application of single-cell sequencing technology in AML, 21 cell types in the bone marrow samples of AML patients were identified, of which six were malignant (van Galen et al., 2019). In addition, it suggested that the CTCs of AML were associated with specific genetic mutation types and different prognoses (van Galen et al., 2019). Therefore, it seemed feasible to construct a novel AML prognostic score based on the CTCs, and how the CTC-based prognostic score would perform remained to be further studied. Experimental methods to acquire the CTCs of samples, including flow cytometry (FCM) (Adan et al., 2017) and single-cell RNA sequencing (scRNA-seq) (Potter., 2018), are costly and infeasible with a large sample size at present. Luckily, increasing computational methods have been developed to infer the CTCs through bulk GEPs (Avila Cobos et al., 2018)—for example, CIBERSORT uses the support vector regression algorithm to deconvolute the bulk GEPs into CTCs based on a reference matrix that comprises the gene expression signatures (GES) of cell types of interest (Newman et al., 2015).

In this study, we aimed to develop a novel prognostic model for *de novo* AML in adults based on the CTCs of patients at diagnosis. Firstly, we constructed a cell type-specific GES reference matrix by conducting a differential expression analysis using AML scRNA-seq profiles. Then, we deconvoluted the bulk GEPs of two AML datasets to CTCs based on the custom reference matrix. Finally, we constructed and evaluated an AML prognostic model, termed CTC score, based on the estimated CTCs. The CTC score showed a comparable performance to previous gene expression-based prognostic models and could act as an independent prognostic factor for AML. In addition, we demonstrated that the CTC score provided additional prognostic information different from LSC17 and APS.

## Materials and Methods

### Data Sources

We downloaded a scRNA-seq dataset, GSE116256 (van Galen et al., 2019), and two bulk gene expression datasets, GSE6891 (Verhaak et al., 2009) and TCGA-LAML (Cancer Genome Atlas Research et al., 2013), for AML from the Gene Expression Omnibus (GEO) data repository (RRID:SCR_005012) and Genomic Data Commons data portal (RRID:SCR_014514), respectively. The scRNA-seq dataset contains single-cell GEPs and cell annotations of 30,712 cells and 27,899 genes from the bone marrow aspirates of 16 AML patients. The cell annotations comprise information such as the number of unique molecular identifiers (UMIs), the number of expressed genes, and the inferred cell type for each cell. A total of 21 cell types were defined, including HSC, HSC-like, progenitor (Prog), Prog-like, granulocyte–monocyte–progenitor (GMP), GMP-like, promonocyte (ProMono), ProMono-like, monocyte (Mono), Mono-like, conventional dendritic cell (cDC), cDC-like, plasmacytoid dendritic cell (pDC), early erythroid progenitor (earlyEry), late erythroid progenitor (lateEry), progenitor B cell (proB), mature B cell (B), plasma cell (plasma), naïve T cell (T), cytotoxic T lymphocyte (CTL), and natural killer cell (NK). Details of the scRNA-seq dataset can be learned from elsewhere (van Galen et al., 2019). For the bulk gene expression dataset GSE6891, 537 GEPs of AML patients profiled by microarray were obtained. For TCGA-LAML, 151 GEPs with fragments per kilobase million normalization were downloaded. The corresponding clinical characteristics and survival information for each sample were downloaded from the cBioPortal database (RRID:SCR_014555).

### Study Design

**FIGURE 1:**
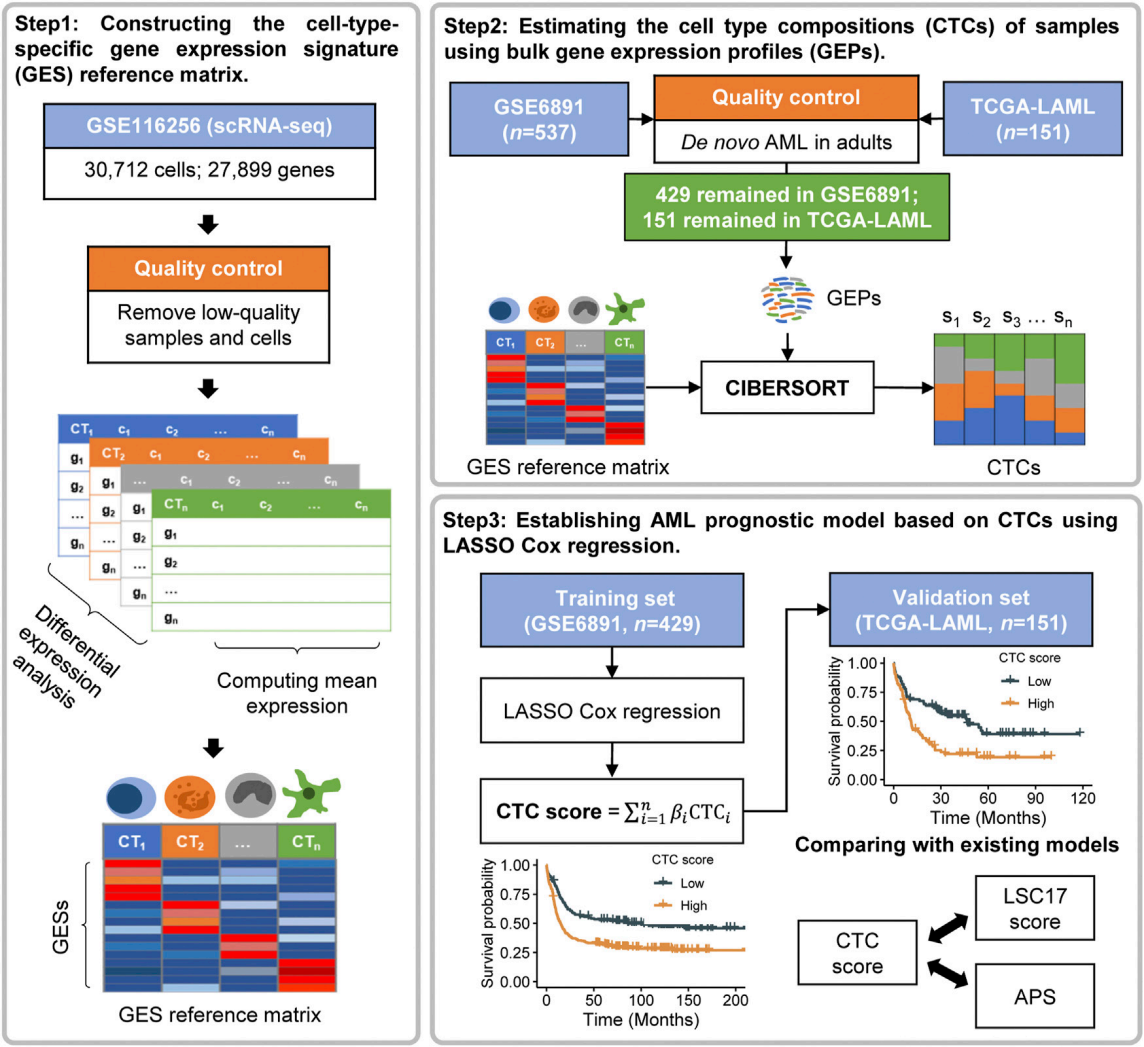
Overview of the workflow.

The workflow of this study is illustrated in Figure 1. We first constructed the GES reference matrix of the 21 cell types required in CIBERSORT (Newman et al., 2015) (RRID:SCR_016955) using AML scRNA-seq profiles. The CTCs of patients in the bulk gene expression datasets of GSE6891 and TCGA-LAML were subsequently estimated. A CTC-based prognostic model was established, with GSE6891 as the training set, and was validated in TCGA-LAML subsequently.

### Data Preprocessing and Quality Control

For the scRNA-seq dataset GSE116256, we excluded cells derived from samples of AML314, AML371, AML722B, and AML997 due to the unconfident cell type annotations (van Galen et al., 2019). Next, we computed the ratio of UMI counts to the number of expressed genes for each cell, termed UTG ratio below. In each cell type, cells with outlier values of UTG ratio were prone to be low in quality. The threshold to filter such cells was determined to be the median UTG ratio plus–minus three times the median absolute deviation (Leys et al., 2013). A total of 27,023 cells remained (Supplementary Figure S1).

The bulk GEPs of GSE6891 were generated by Affymetrix Human Genome U133 Plus 2.0 Array (Verhaak et al., 2009). The raw CEL files were processed using affy (version 1.66.0) and normalized by the Gene Chip Robust Multi-array Average (Wu et al., 2004) algorithm using gcrma (version 2.60.0) Bioconductor R package. The probe set IDs were transformed to the corresponding gene symbols according to the chip definition file (GEO accession: GPL570). The probe sets that did not match any gene symbols or matched multiple gene symbols were filtered out. To retain enough genes for subsequent analysis, we computed the mean expression of probe sets that matched the same gene and chose the probe set with the highest average gene expression to represent that gene (Ng et al., 2016). Among the cases in GSE6891, we only retained *de novo* AML cases whose age at diagnosis were greater or equal to 18 with completed survival information.

For TCGA-LAML, the ensemble gene IDs of the downloaded GEPs were transformed to gene symbols according to the comprehensive gene annotation files of GENCODE release 38 (GRCh38.p13; RRID:SCR_014966) in gene transfer format. We filtered out the ensemble gene IDs matching the same gene symbol due to the difficulty in determining which ensemble gene ID to represent that gene. Among the cases in TCGA-LAML, we took the same filtering criteria as implemented for the GSE6891 dataset.

### Constructing the Cell Type-Specific Gene Expression Signatures Reference Matrix

We constructed the cell type-specific GES reference matrix based on the AML scRNA-seq GEPs using Seurat (Stuart et al., 2019) (version 3.2.0) R package. First, the single-cell GEPs of AML patients were integrated and imported into a Seurat object. All cells were labeled as the cell type in the annotation file. Then, we normalized the UMI counts to counts per million (CPM) and performed natural-log transformation [log (CPM+1)]. Subsequently, we conducted the differential expression analysis using FindAllMarkers function to acquire highly expressed genes of each cell type by comparing the cells of 1 cell type against all others in turn. The tests of comparisons between groups used the “bimod” method, a likelihood-ratio test for single-cell gene expression (McDavid et al., 2013). The “min.pct” parameter was set to 0. Other parameters were set as default. The acquired highly expressed genes of each cell type with the adjusted *p*-values lower than 0.05 and the average natural-log fold-change (logFC) above 1 were retained (Supplementary Figure S2 and Supplementary Table S1). Notably, the highly expressed genes selected to build the GES reference matrix are the dominant influence factor for CTC estimations, thereby affecting subsequent modeling. Therefore, we extracted the top 25, 50, 100, and 150 most significantly highly expressed genes for each cell type and computed the mean expression by cell type to build 4 cell type-specific GES reference matrices (GES25, GES50, GES100, and GES150; Supplementary Figure S3, Supplementary Table S2, Supplementary Table S3, Supplementary Table S4, and Supplementary Table S5) (Donovan et al., 2020).

### Simulations to Examine the Accuracy of CIBEROSRT and Gene Expression Signatures Matrices

We performed a simulation analysis to examine the accuracy of CIBERSORT using the custom cell type-specific GES reference matrices. Specifically, we first generated 100 artificial samples using scRNA-seq profiles. For each sample, we selected a random number of cells for each cell type from at least 50 to the maximum number of cells for that cell type through the cell barcodes (Donovan et al., 2020). The normalized GEPs of these cells were summed to create the artificial sample with known cell type compositions. Subsequently, we ran CIBERSORT on these artificial samples using different GES matrices. Additionally, two other deconvolution methods, MuSiC (Wang et al., 2019) and MOMF (Sun et al., 2019), were also used for comparisons. The Pearson correlation coefficients of the real proportions and the estimated proportions were computed by each cell type as the metric of accuracy.

### Estimating Cell Type Compositions Using CIBERSORT

The simulation results showed that the performances of CIBERSORT, MuSiC, and MOMF were similar (Supplementary Figure S4). However, we noticed that MuSiC and MOMF took a much longer running time and much more memory consumptions (data not shown). Accordingly, we chose CIBEROSRT to estimate the relative proportions of 21 AML cell types for the bulk gene expression datasets GSE6891 and TCGA-LAML, setting 100 permutations and disabling the quantile normalization option.

### Constructing an Acute Myeloid Leukemia Prognostic Model Based on Cell Type Compositions

After estimating the CTCs (Supplementary Table S6, Supplementary Table S7, Supplementary Table S8, and Supplementary Table S9), we found that the estimated proportions of some cell types were almost 0 for most of the samples, probably due to estimation error. To reduce the influence on subsequent modeling, we converted the cell types whose mean proportions were lower than 0.05 or median proportions were equal to 0 to dichotomous variables, with 0 as the cutoff value. Cell types converted to dichotomous or that remained continuous in both datasets and whose Pearson correlation coefficient was *r* > 0.8 in the simulations were used to train and validate the prognostic model.

The bulk gene expression dataset GSE6891 was set as the training set, and TCGA-LAML was set as the validation set to establish and validate a novel prognostic model for AML based on CTCs. With OS as the survival outcome, we performed the least absolute shrinkage and selection operator (LASSO) Cox regression (Simon et al., 2011) and 10-fold cross-validation using glmnet (version 4.1–1) R package. To obtain a robust model, we repeated this process 100 times using different random seeds, and cell types with non-zero coefficients in at least 95 fittings were retained. The coefficients of 100 fitting processes for the retained cell types were averaged as the final coefficient (Elsayed et al., 2020). The linear combination of the selected cell types in the LASSO Cox regression model weighted by the coefficients served as the prognostic marker for AML, called CTC score. For better interpretation and visualization, we partitioned all patients into low- and high-CTC-score groups by median.

The established CTC score was validated in TCGA-LAML. We computed the CTC scores for patients in TCGA-LAML based on the linear equation above (Supplementary Table S10). We likewise partitioned the patients in the validation set into low- and high-CTC-score groups based on the median. Kaplan–Meier curves were used to display the different prognoses between low- and high-CTC-score groups.

We considered displaying the CTC score established on the CTCs estimated with GES100 as the reference matrix to be the main results. Other prognostic models based on the CTCs estimated using GES25, GES50, and GES150 were considered as sensitivity analysis and could be accessible in Supplementary Figure S5. The Harrell’s concordance index (C-index) was used to compare the performance of these models (Harrell et al., 1996).

### Verifying the Prognostic Independence of the Cell Type Composition Score

We found that GMP-like has a great weight when computing the CTC score (see results part). It has been reported that GMP-like is highly associated with two abnormal karyotypes (i.e., *PML-RARA* and *RUNX1-RUNX1T1*), both of which indicate a favorable prognosis (Appelbaum et al., 2006; Wang and Chen., 2008; van Galen et al., 2019). Thus, it is crucial to verify whether the prognostic significance of CTC score was dominantly captured by existing prognostic factors such as karyotypes and cytogenetic risk classifications. To verify this point, we first implemented univariable Cox regressions for clinical characteristics. The clinical characteristics significant in both training and validation dataset and CTC score were introduced to multivariable Cox regressions using survival (version 3.2–7) R package.

### Comparing the Cell Type Composition Score with the LSC17 Score and Acute Myeloid Leukemia Prognostic Score

We further evaluated the performance of CTC score by comparing it with the LSC17 score and APS. The LSC17 score was constructed by the expression of 17 genes highly expressed in LSCs, while the APS was constructed by the expression of 16 genes acquired by LASSO Cox regression (Ng et al., 2016; Docking et al., 2021). The LSC17 score and APS for patients in the validation set TCGA-LAML were computed in compliance with the data processing flow and calculation equation according to the original articles (Supplementary Table S10) (Ng et al., 2016; Docking et al., 2021). Considering the comparability, all three prognostic scores were not converted to dichotomous variables. We implemented the time-dependent receiver operating characteristic (ROC) curve analysis to evaluate and compare the predictive accuracy using area under the ROC curve (AUC) as the indicator. The predictive sensitivities and specificities of CTC score, LSC17 score, and APS at 1-, 2-, 3-, and 5-years timepoints were computed and compared using timeROC (Blanche et al., 2013) (version 0.4) R package.

### Statistical Analysis

For the clinical characteristics of patients in the bulk gene expression datasets GSE6891 and TCGA-LAML, continuous variables were described by medians and ranges, and categorical variables were described by frequencies and proportions. We used the Wilcoxon test or Kruskal–Wallis test for group comparisons of continuous variables and the chi-square test or Fisher’s exact test for that of categorical variables. All statistical tests were two-tailed, and *p*-values lower than 0.05 were considered statistically significant. All the analyses were performed in R-4.0.2.

## Results

### Clinical Characteristics and Cell Type Compositions for Two Bulk Acute Myeloid Leukemia Datasets

For the bulk gene expression dataset GSE6891, 11 patients whose age at diagnosis was lower than 18, 17 patients of myelodysplastic syndrome, and four patients with missing survival information were filtered out. Eventually, 429 patients were eligible, whereas all patients in TCGA-LAML passed the filtering criteria. The descriptive characteristics of patients in these two datasets are shown in Table 1. Patients in GSE6891 were younger than those in TCGA-LAML (*p* < 0.0001). FAB classification (*p* = 0.0010) and cytogenetic risk (*p* = 0.0313) were also different between GSE6891 and TCGA-LAML. Patients in GSE6891 comprises more FAB-M5 subtype (23.3% in GSE6891 *vs* 9.9% in TCGA-LAML) and less poor cytogenetic risk strata (19.3% in GSE6891 *vs* 23.8% in TCGA-LAML). The CTCs for patients in the GSE6891 and TCGA-LAML datasets estimated with GES100 as the reference matrix are shown in Supplementary Figure S6.

**TABLE 1:** Characteristics of acute myeloid leukemia patients in the training set and the validation set.

Characteristic	GSE6891	TCGA-LAML	*p*-value
	(Training set; *n* = 429)	(Validation set; *n* = 151)	
**Age at diagnosis, years**			<0.0001
Median (range)	44 (18–60)	56 (21–88)	
≤55	361 (84.1)	74 (49.0)	
>55	68 (15.9)	77 (51.0)	
**Sex**			0.3233
Female	218 (50.8)	69 (45.7)	
Male	211 (49.2)	82 (54.3)	
**FAB classification**			0.0010
M0	16 (3.7)	15 (9.9)	
M1	94 (21.9)	36 (23.8)	
M2	100 (23.3)	37 (24.5)	
M3	24 (5.6)	15 (9.9)	
M4	81 (18.9)	29 (19.2)	
M5	100 (23.3)	15 (9.9)	
M6	6 (1.4)	2 (1.3)	
M7	0 (0)	1 (0.7)	
NA	8 (1.9)	1 (0.7)	
**Cytogenetic risk**			0.0313
Good	91 (21.2)	31 (20.5)	
Intermediate	245 (57.1)	81 (53.6)	
Poor	83 (19.3)	36 (23.8)	
NA	10 (2.3)	3 (2.0)	
**Karyotype**			0.0964
Others	351 (81.8)	127 (84.1)	
*PML-RARA*	21 (4.9)	14 (9.3)	
*RUNX1-RUNX1T1*	32 (7.5)	7 (4.6)	
NA	25 (5.8)	3 (2.0)	

Patients with missing values were removed before performing the statistical tests. Chi-square tests were implemented to compare the constituent ratios of characteristics between the training set GSE6891 and the validation set TCGA-LAML, except for FAB classification, for which Fisher’s exact test was conducted.

FAB, French–American–British; NA, not available; CTC, cell type composition.

### Cell Type Composition-Based Prognostic Score for Acute Myeloid Leukemia

The median follow-up time of patients in the bulk gene expression datasets GSE6891 and TCGA-LAML was 20.11 months [interquartile range (IQR), 7.89–92.78 months] and 19 months (IQR, 6.45–42.1 months), respectively. We fitted a LASSO Cox regression model and defined the CTC score computed by the following equation:

CTC score = (−1.7016 × GMP-like) + (0.2015 × HSC-like) + (−0.293 × T), where HSC-like and T were dichotomous. The negative coefficient of GMP-like indicated that lower relative proportions of GMP-like at diagnosis would predict worse survival outcomes. The estimated HSC-like greater than 0 and T equal to 0 would predict worse prognoses.

**FIGURE 2:**
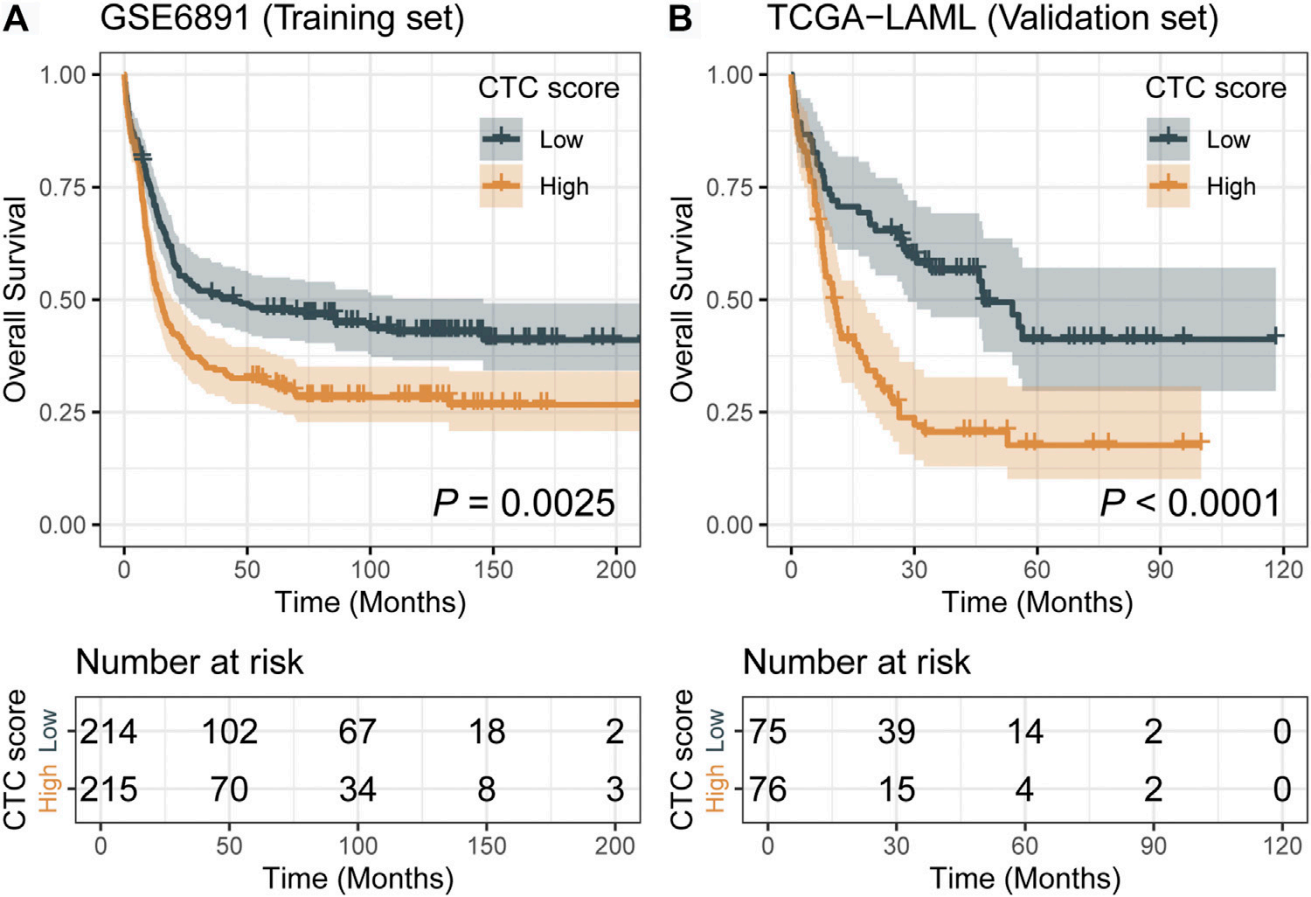
Kaplan–Meier curves of low-cell type composition (CTC)-score and high-CTC-score groups for the training set GSE6891 **(A)** and the validation set TCGA-LAML **(B)**.

Comparing with the low-CTC-score group, the high-CTC-score group showed a 1.57-fold (95% CI, 1.23 to 2.00; *p* = 0.0002) higher overall mortality risk in the training set GSE6891 and 2.32-fold (95% CI, 1.53 to 3.51; *p* < 0.0001) in the validation set TCGA-LAML (Table 2). The 5-years OS rate for GSE6891 was 47.7% (95% CI, 41.4–54.9%) in the low-CTC-score group and 31.1% (95% CI, 25.5–37.9%) in the high-CTC-score group. For TCGA-LAML, the 5-years OS rate was 41.2% (95% CI, 29.7–57.1%) and 17.7% (95% CI, 10.2–30.7%) in the low-CTC-score group and high-CTC-score group, respectively (Figure 2).

**TABLE 2:** Univariable Cox regression with overall survival as the outcome.

Characteristic	GSE6891 (Training set, *n* = 429)		TCGA-LAML (Validation set, *n* = 151)	
	HR (95% CI)	*p*-value	HR (95% CI)	*p*-value
**Age at diagnosis, years**				
≤55	Reference		Reference	
>55	1.83 (1.36–2.47)	<0.0001	2.71 (1.79–4.11)	<0.0001
**Sex**				
Female	Reference		Reference	
Male	0.94 (0.74–1.19)	0.6002	1.01 (0.68–1.51)	0.9465
**FAB classification**				
M0	2.14 (0.96–4.79)	0.0632	3.76 (1.18–12.04)	0.0256
M1	1.43 (0.75–2.72)	0.2770	3.73 (1.29–10.81)	0.0152
M2	1.40 (0.74–2.66)	0.3046	3.33 (1.15–9.64)	0.0262
M3	Reference		Reference	
M4	1.28 (0.67–2.47)	0.4574	3.93 (1.34–11.53)	0.0126
M5	1.66 (0.88–3.14)	0.1186	4.57 (1.42–14.67)	0.0106
M6	0.89 (0.25–3.18)	0.8532	9.69 (1.74–53.99)	0.0095
M7	NA	NA	7.83 (0.86–71.13)	0.0675
**Cytogenetic risk**				
Good	Reference		Reference	
Intermediate	1.99 (1.39–2.84)	0.0002	3.11 (1.58–6.10)	0.0010
Poor	3.40 (2.27–5.10)	<0.0001	4.36 (2.10–9.03)	<0.0001
**Karyotype**				
Others	Reference		Reference	
*PAML-RARA*	0.39 (0.18–0.82)	0.0136	0.28 (0.10–0.78)	0.0143
*RUNX1-RUNX1T1*	0.39 (0.22–0.70)	0.0017	0.45 (0.14–1.44)	0.1800
**CTC score**				
Low	Reference		Reference	
High	1.57 (1.23–2.00)	0.0002	2.31 (1.53–3.51)	<0.0001

Patients with missing values were removed before performing the statistical tests.

HR, hazard ratio; CI, confidence interval; FAB, French–American–British; NA, not available; CTC, cell type composition.

The individual-level results of CTCs estimated using GES25, GES50, GSE100, and GES150 could be obtained in Supplementary Table S6, Supplementary Table S7, Supplementary Table S8, and Supplementary Table S9. As displayed in Supplementary Figure S5, the CTC-based scores established by reference matrices with different GES matrices were robustly associated with the OS of AML in the validation set, with C-index ranging from 0.64 (95% CI, 0.58–0.70) to 0.67 (95% CI, 0.61–0.73).

### Cell Type Composition Score Is an Independent Factor in Predicting Acute Myeloid Leukemia Prognosis

**FIGURE 3:**
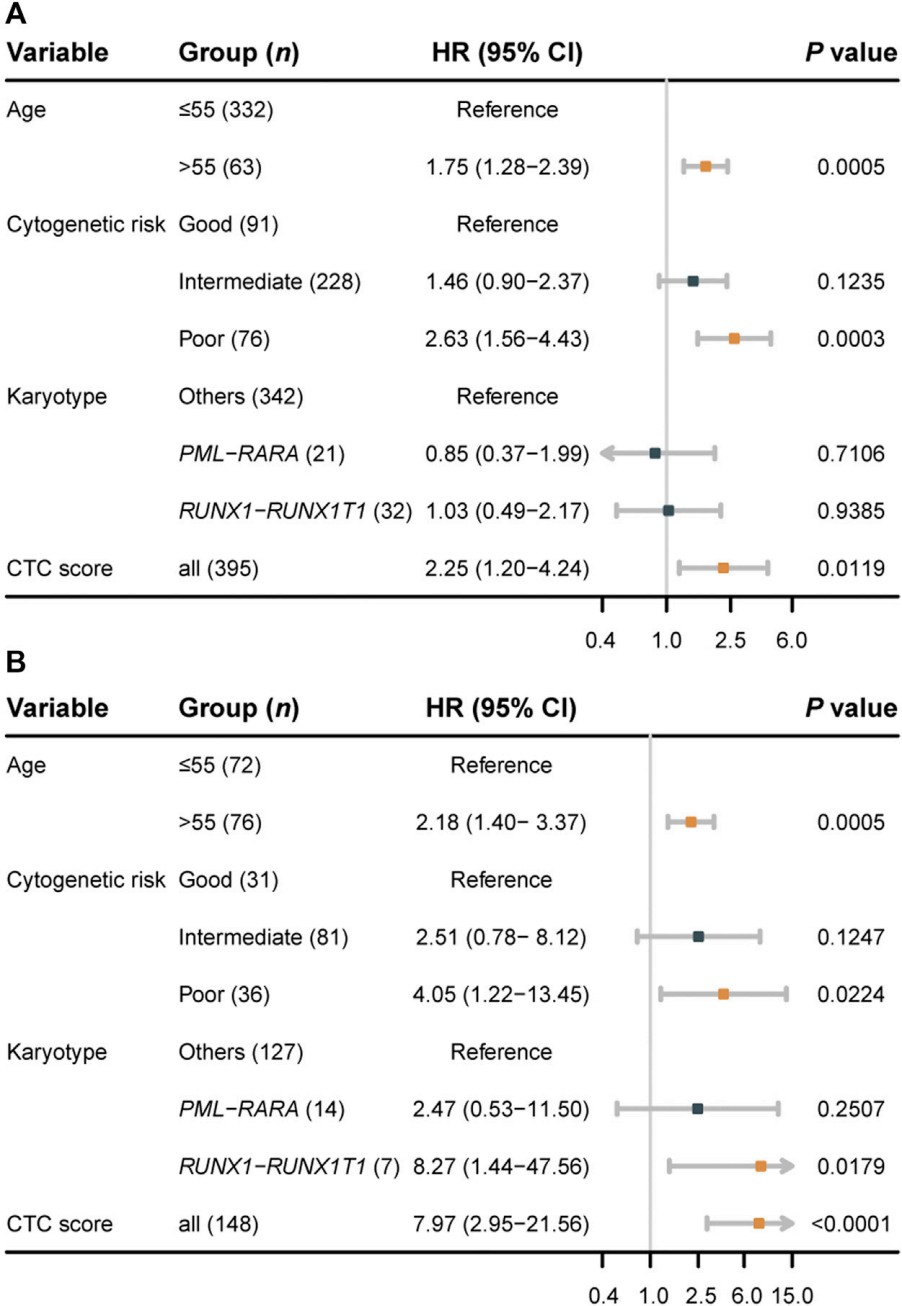
Forest plots of multivariable Cox regression results for the training set GSE6891 **(A)** and the validation set TCGA-LAML **(B)**.

We performed univariable and multivariable Cox regressions in both the training and validation sets to test whether the CTC score is an independent factor associated with the OS for AML in adults. Among the clinical characteristics, age at diagnosis, cytogenetics risk, and karyotype were significantly associated with OS in both datasets (Table 2). The multivariable Cox regression results showed that CTC score remained statistically significant in GSE6891 (HR = 2.25; 95% CI, 1.20 to 4.24; *p* = 0.0119) and TCGA-LAML (HR = 7.97; 95% CI, 2.95 to 21.56; *p* < 0.0001) when adjusting for age at diagnosis, cytogenetic risk, and karyotype (Figure 3). These results suggested that CTC score can predict the prognosis of AML independent of age at diagnosis, cytogenetic risk, and karyotype.

### Cell Type Composition Score Provides Additional Prognostic Information Different from LSC17 and Acute Myeloid Leukemia Prognostic Score

**FIGURE 4:**
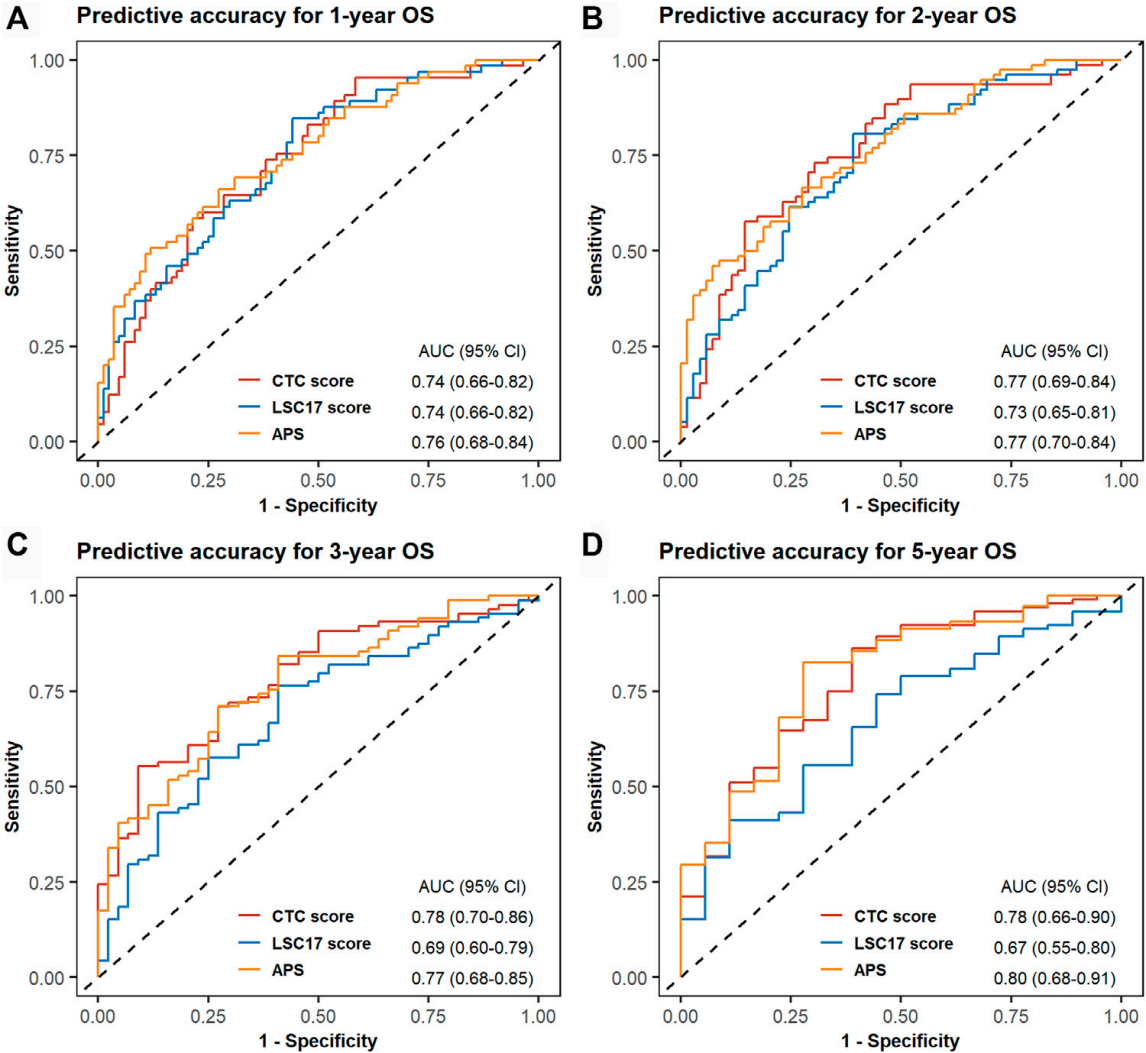
Time-dependent receiver operating characteristic (ROC) curves for cell type composition score, LSC17 score, and acute myeloid leukemia prognostic score of the validation set TCGA-LAML. One-year **(A)**, 2-years **(B)**, 3-years **(C)**, and 5-years **(D)** ROC curves and the corresponding areas under ROC curve with 95% CI are displayed.

**FIGURE 5:**
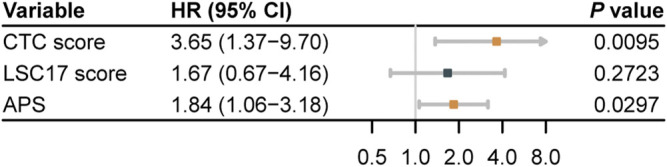
Forest plot for multivariable Cox regression, incorporating three acute myeloid leukemia prognostic scores of the validation set TCGA-LAML.

In TCGA-LAML, we evaluated the predictive accuracy of 1-, 2-, 3-, and 5-years OS using ROC curves. The corresponding AUCs and 95% CIs for CTC score, LSC17 score, and APS were computed as shown in Figure 4. The differences in AUCs of CTC score *versus* LSC17 score and CTC score *versus* APS at four time points were not statistically significant (Supplementary Table S11), suggesting that CTC score can achieve a similar predictive accuracy compared with LSC17 score and APS. Additionally, we simultaneously included CTC score, LSC17 score, and APS into the multivariable Cox regression (Figure 5). CTC score (HR = 3.65; 95% CI, 1.37 to 9.7; *p* = 0.0095) and APS (HR = 1.84; 95% CI, 1.06 to 3.18; *p* = 0.0297) remained statistically significant, suggesting that both CTC score and APS could capture additional prognostic information compared with LSC17 score. Furthermore, the additional prognostic information captured by the CTC score was different from that captured by APS.

## Discussion

In the present study, we have constructed an AML prognostic score based on the assumption that the CTCs of AML patients at diagnosis can reflect the genetic abnormalities and are thus correlated with their prognosis (van Galen et al., 2019). To estimate CTCs, we first constructed a cell type-specific GES reference matrix GES100 through a differential expression analysis of the AML scRNA-seq dataset. Then, we applied the CIBERSORT algorithm to deconvolute the bulk GEPs of AML samples to CTCs by the custom GES reference matrix. Subsequently, an AML prognostic score based on the CTCs (i.e., CTC score) comprising 3 cell types, GMP-like, HSC-like, and T, was established for *de novo* AML in adults. CTC score was significantly associated with the OS in both the training set and the validation set.

Previous studies applying CIBERSORT to estimate the immune microenvironment for AML all used LM22, which contains the GESs of 22 immunocytes provided by the author as the reference matrix (Newman et al., 2015; Xu et al., 2020; Cheng et al., 2021; Jia et al., 2021). However, the estimates of CTCs might be inaccurate in these studies because of the resemblance between normal immunocytes and malignant leukemic blasts, especially for the myeloid lineages—for example, both Xu et al. (2020) and Cheng et al. (2021) identified that higher relative proportions of M2 macrophage were associated with a poorer prognosis for AML. Additionally, Xu et al. (2020) suggested the marker gene of M2 macrophage CD206, also presenting in immature dendritic cells (DCs) (Wollenberg et al., 2002), as a novel prognostic predictor. However, we found that CD206 was highly expressed in cDC-like (Supplementary Figure S7). Thus, the estimated proportions for M2 macrophage might be overestimated due to the similarity between cDC-like and M2 macrophage when using LM22 as the reference. To fix this issue, we constructed custom GES reference matrices containing all 21 cell types of the bone marrow annotated by the single-cell GEPs. In this manner, the estimated relative proportions of using CIBERSORT could reflect the real proportions of each cell type in the sample. When considering both the normal and the malignant cell types in AML samples, the established CTC score showed a powerful prognostic significance.

We noticed that the coefficient of GMP-like in CTC score was greater than the other 2 cell types. It has been revealed before that GMP-like was associated with PML-RARA and RUNX1–RUNX1T1 fusion in the TCGA-LAML dataset (van Galen et al., 2019). This finding was repeated in the bulk gene expression dataset GSE6891 (Supplementary Figure S8). Researchers found that the PML–RARA fusion leads to a block in the differentiation of myeloid cells at the promyelocytic stage (Grisolano et al., 1997). In recent decades, the PML–RARA fusion-induced AML has become highly curable since the broad application of target chemotherapy drugs, all-trans retinoic acid and arsenic trioxide, into clinical use (Wang and Chen., 2008). The RUNX1–RUNX1T1 fusion-induced AML has also been determined to have a good prognosis (Appelbaum et al., 2006). It is characterized by the expressed myeloperoxidase, a protein expressed mainly in neutrophils, in more than 90% of leukemia blasts (Schlaifer et al., 1993; Aratani., 2018). Both of these two gene fusions are considered to be of good prognosis in cytogenetic risk classification (Slovak et al., 2000). In other words, the CTC score is probably confounded by these two gene fusions for the great weight of GMP-like. Analogously, other covariates imbalanced such in the training and validation sets as the cytogenetic risk might also confound the results. Therefore, it is crucial to figure out whether the CTC score can provide additional and independent prognostic information to AML prognosis in comparison to the existing classifications. In our study, we have justified this by conducting multivariable Cox regression analyses. We introduced age at diagnosis, karyotype, and cytogenetic risk as covariates for both the training and validation datasets, and the CTC score remained statistically significant.

Except for the LSC17 score and APS, most of the existing studies were based on transcriptomic profiles aiming to construct prognostic scores or find genes associated with the prognosis of AML in adults or pediatric AML were based on transcriptomic profiles (Duployez et al., 2019; Huang et al., 2019; Elsayed et al., 2020; Wang et al., 2020). Some of the genes in these models were inexplicable. Few AML prognostic studies focused on the CTCs of samples from AML patients at diagnosis. In our study, we showed that the AML prognostic model established on the CTCs could independently assess the overall survival of AML patients. The CTC score achieved comparative performance in predicting AML prognosis compared with the gene expression-based prognostic scores. Furthermore, we found that the CTC score could provide additional information different from the LSC17 score and APS. The CTC score clarified that GMP-like was a powerful cell marker predicting the prognosis for AML. Rapid detection of the proportions of GMP-like in the samples from AML patients at diagnosis was expected to aid prognostic classification in the future. Nevertheless, more datasets are required to further verify the effectiveness of the CTC score. Besides this, to incorporate CTC score, APS, and other prognostic factors into a more powerful prognostic model for AML is expected in further studies.

There exist several limitations in the present study. First, the similarity between different cell types inevitably affects the estimation of CIBERSORT. At present, the highly expressed genes of each cell type are typically obtained by comparing 1 cell type against all others. Such a method makes it difficult to distinguish 1 cell type from another similar cell type, especially when the number of one of the cell types is relatively small. To mitigate this influence, we filtered out highly expressed genes with logFC lower than 1 and chose the most significant for each cell type. Second, the discrepancies of distribution for some cell types (e.g., ProMono-like) between the training set and the validation set, as shown in Supplementary Figure S6, might be caused by estimation error, different composition in AML subtypes between datasets, and different transcriptome sequencing approach. This might limit the power to identify the associations of these cell types with AML prognosis. Third, we assumed that samples from bone marrow aspirates and peripheral blood comprised the same cell types. The samples of bulk GEPs datasets GSE6891 and TCGA-LAML were from different tissues, bone marrow aspirates, or peripheral blood, which might cover the prognostic role of some anti-tumor cell types—for example, T cells accounted for a great part in the single-cell dataset (Supplementary Figure S1), whereas the estimated proportions of bulk datasets were less (Supplementary Figure S6).

In conclusion, our study established a novel AML prognostic score using CTCs for *de novo* AML in adults. CTC score has great potential to assist clinicians to formulate individualized treatment plans, thereby improving the prognosis for AML patients.

## Data Availability

Publicly available datasets were analyzed in this study. This data can be found here: https://www.ncbi.nlm.nih.gov/geo/query/acc.cgiacc=GSE116256, Gene Expression Ominibus, accession number:GSE116256; https://www.ncbi.nlm.nih.gov/geo/query/acc.cgiacc=GSE6891, Gene Expression Ominibus, accession number: GSE6891; https://portal.gdc.cancer.gov/, Genomic Data Commons Data Portal, TCGA-LAML; https://www.cbioportal.org/study/clinicalDataid=laml_tcga_pub, cBioPortal, Acute Myeloid Leukemia (TCGA, NEJM 2013).
