# Supplementary figures and images for: Deconvolution of Bulk Gene Expression Profiles with Single-Cell Transcriptomics to Develop a Cell Type Composition-Based Prognostic Model for Acute Myeloid Leukemia
Source: Front Cell Dev Biol. 2021 Nov 12;9:762260. doi: 10.3389/fcell.2021.762260 (PMC8633313; doi:10.3389/fcell.2021.762260)

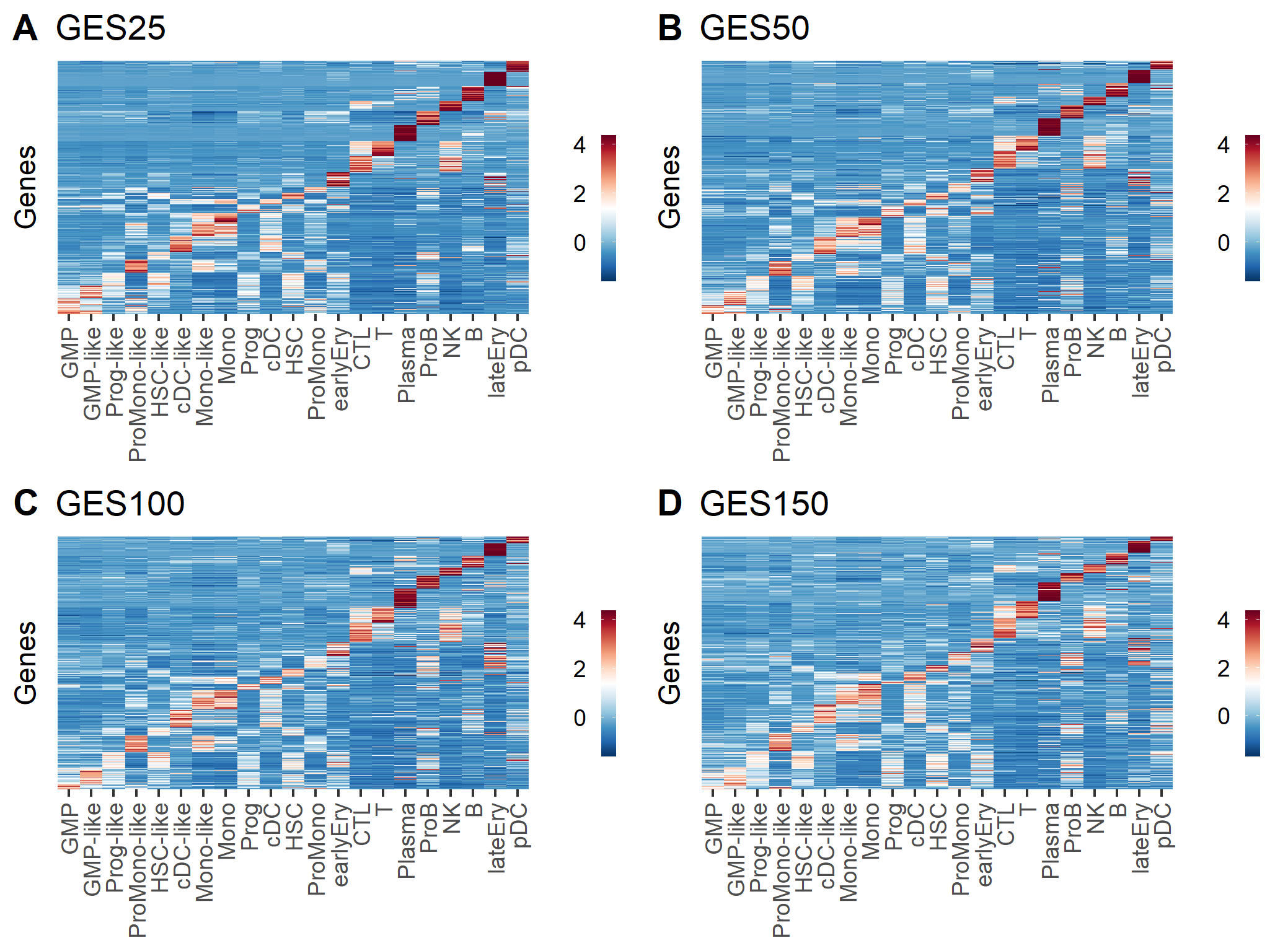

Supplement: Supplementary file 1 [file Image3.TIFF]

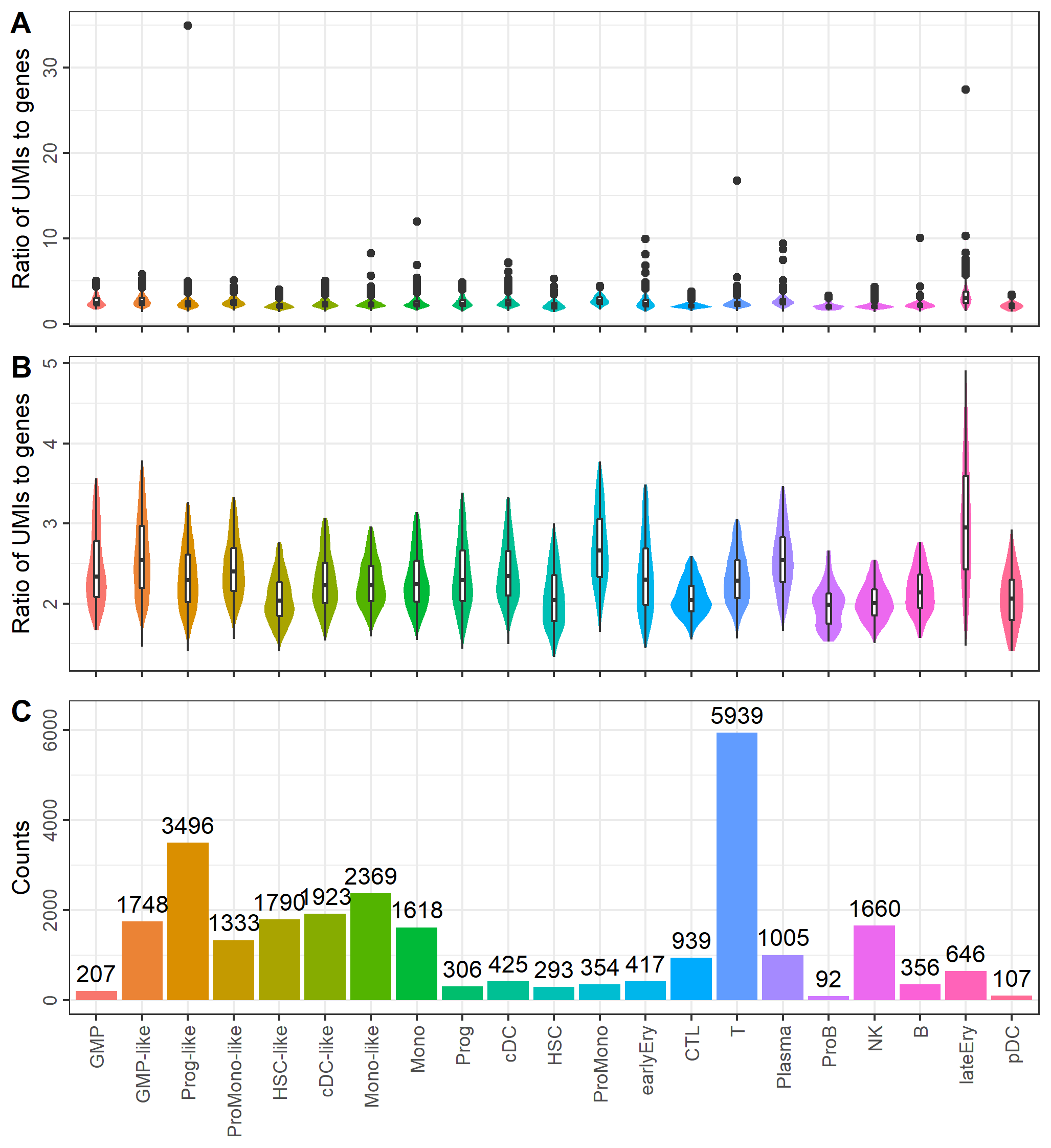

Supplement: Supplementary file 2 [file Image1.TIFF]

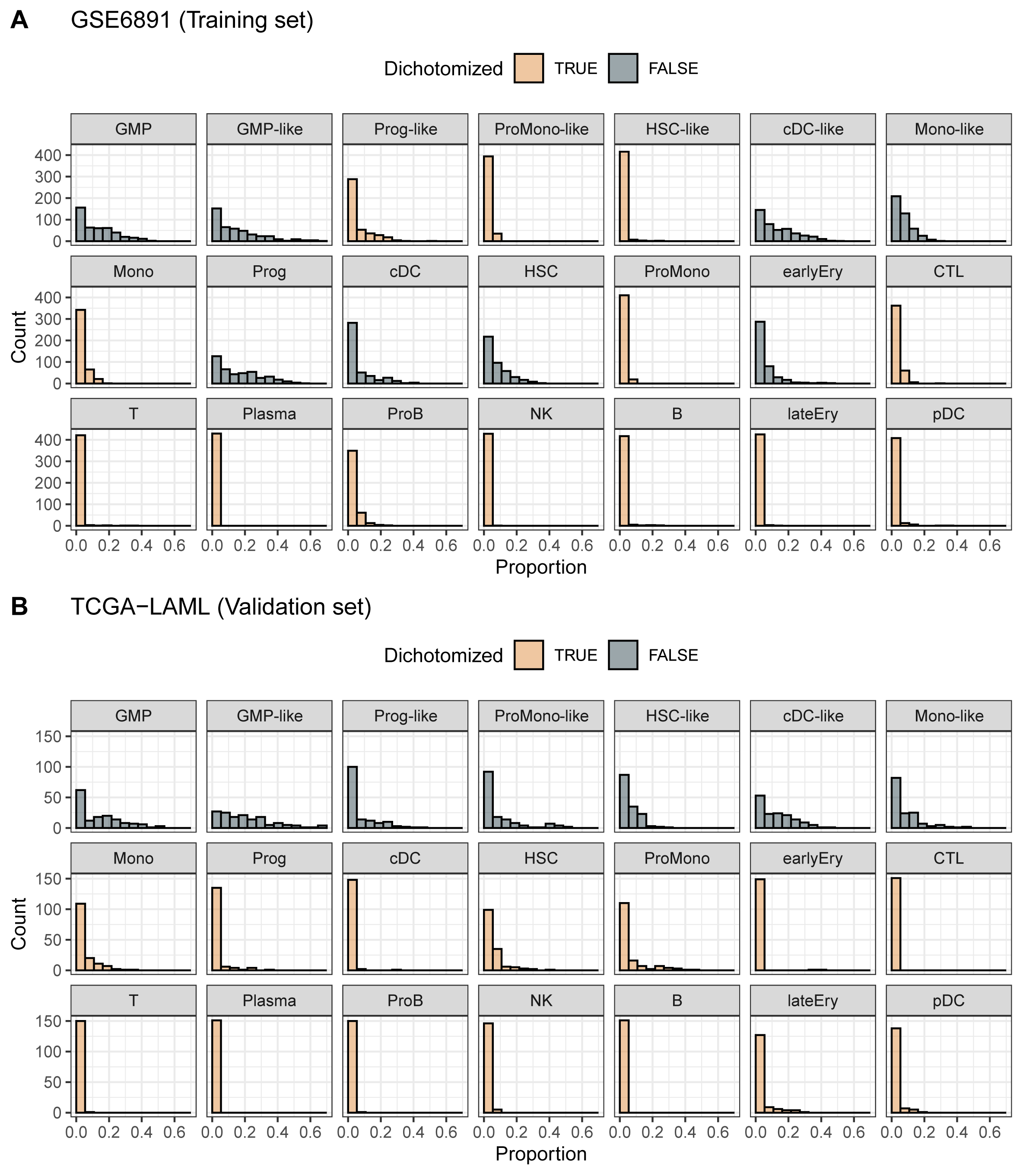

Supplement: Supplementary file 3 [file Image6.TIF]

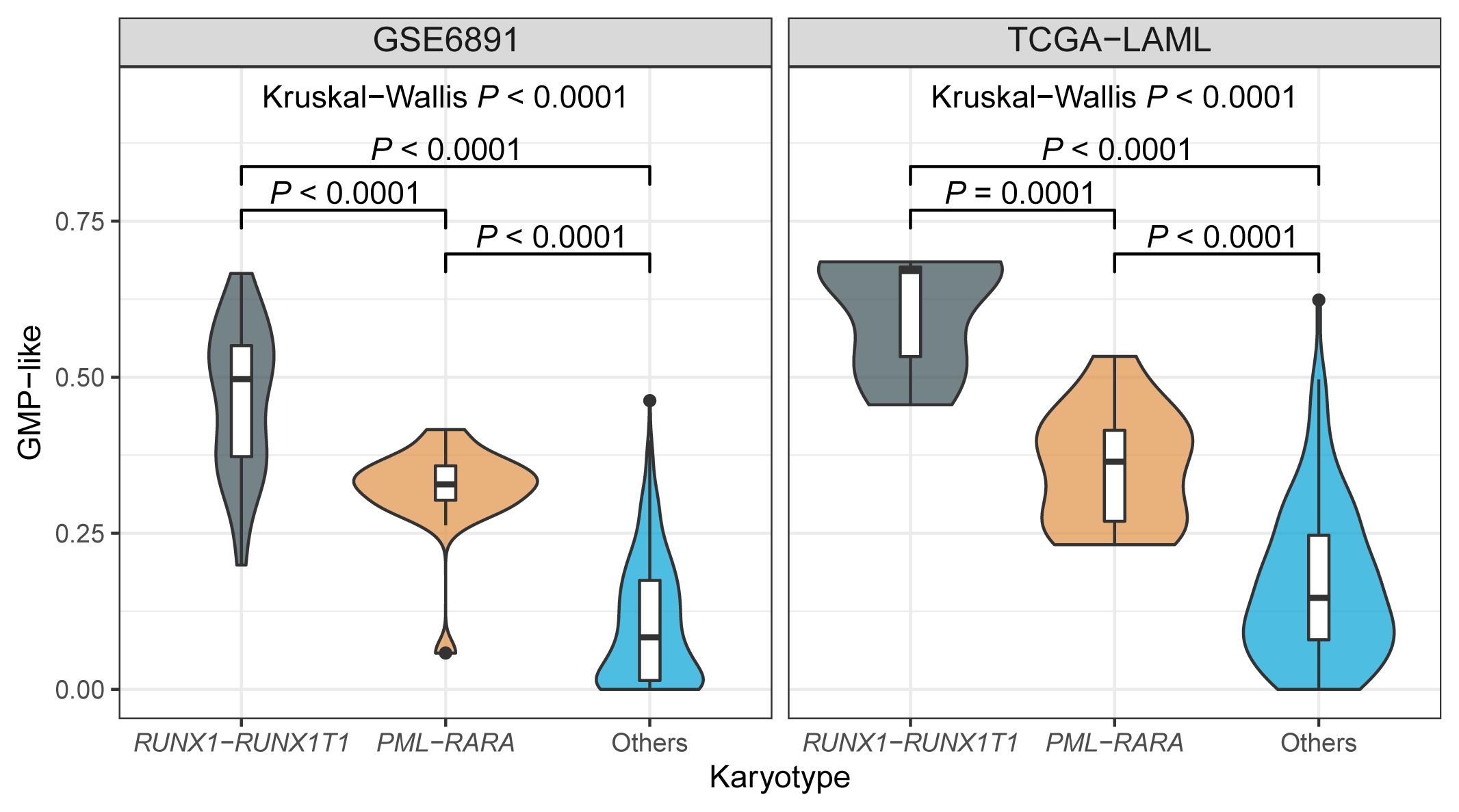

Supplement: Supplementary file 4 [file Image8.TIF]

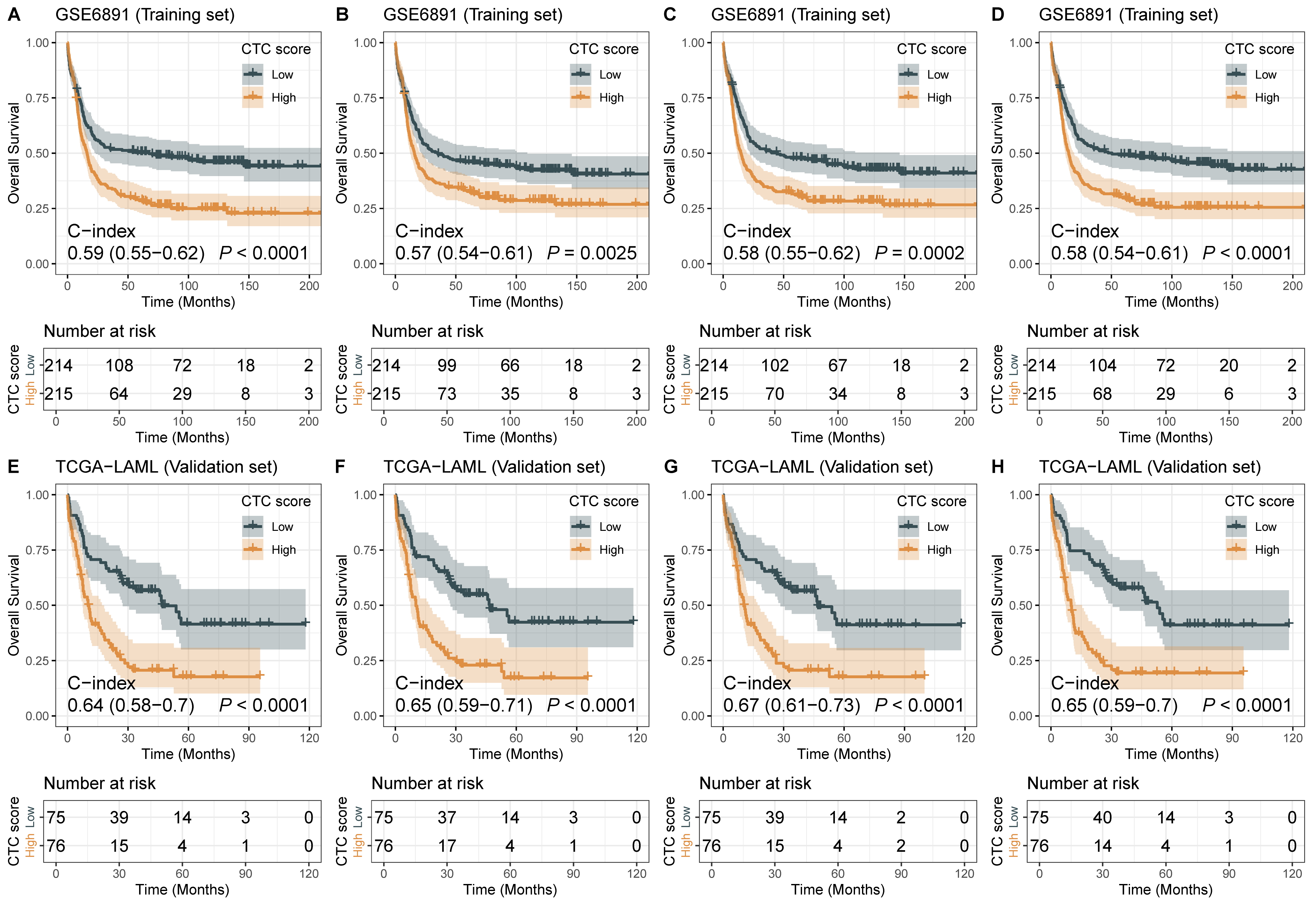

Supplement: Supplementary file 5 [file Image5.TIF]

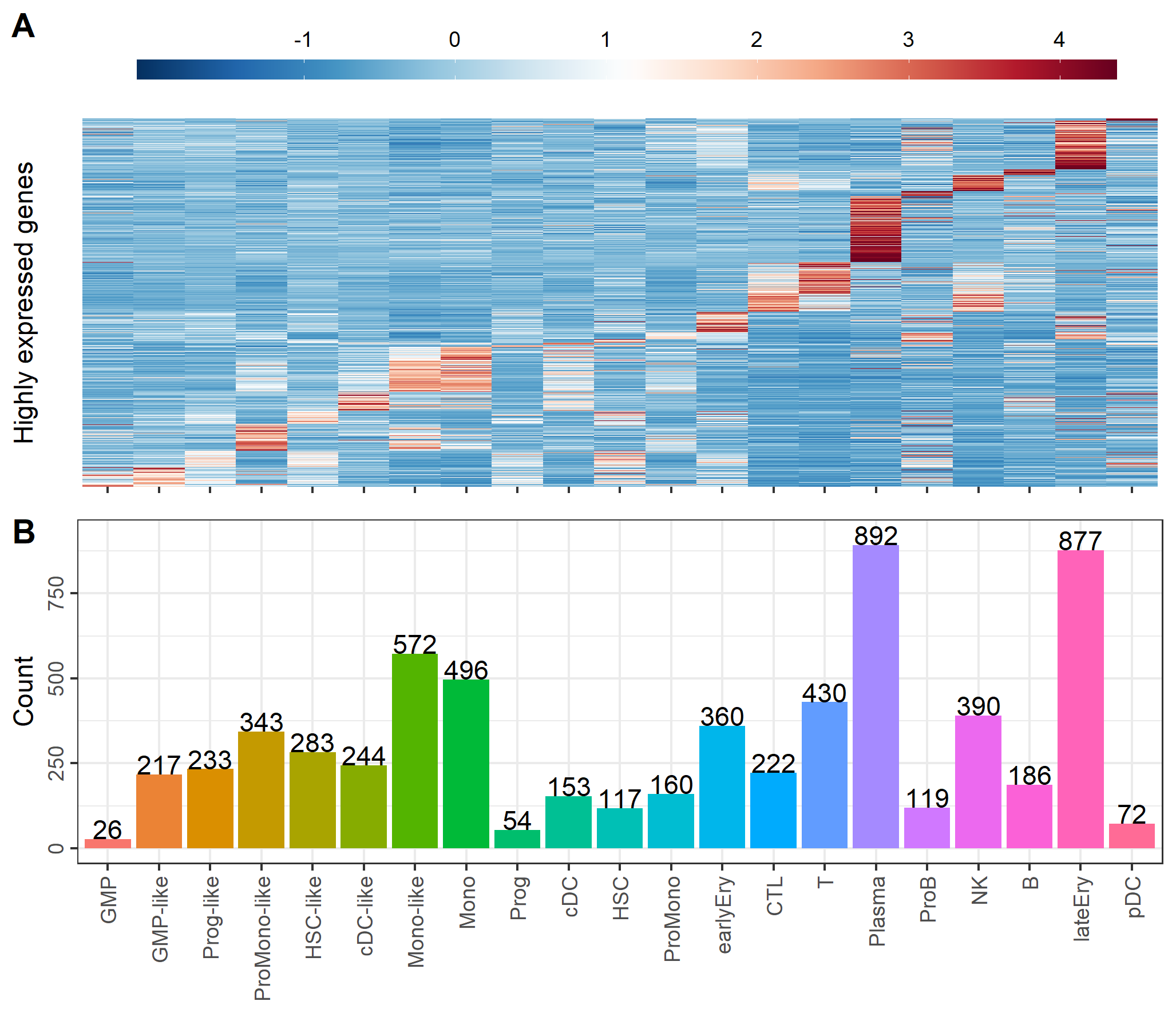

Supplement: Supplementary file 6 [file Image2.TIFF]

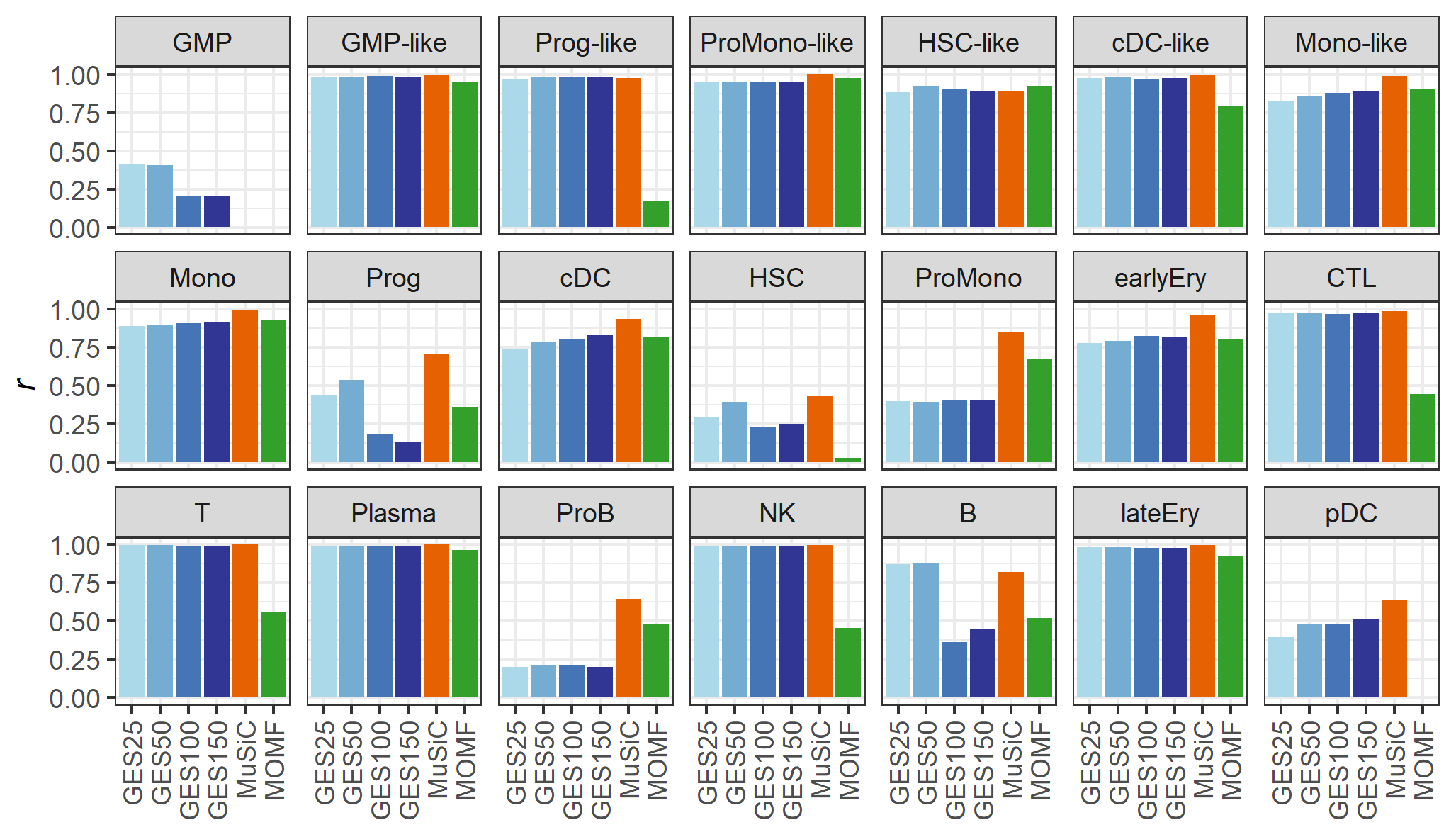

Supplement: Supplementary file 7 [file Image4.TIFF]

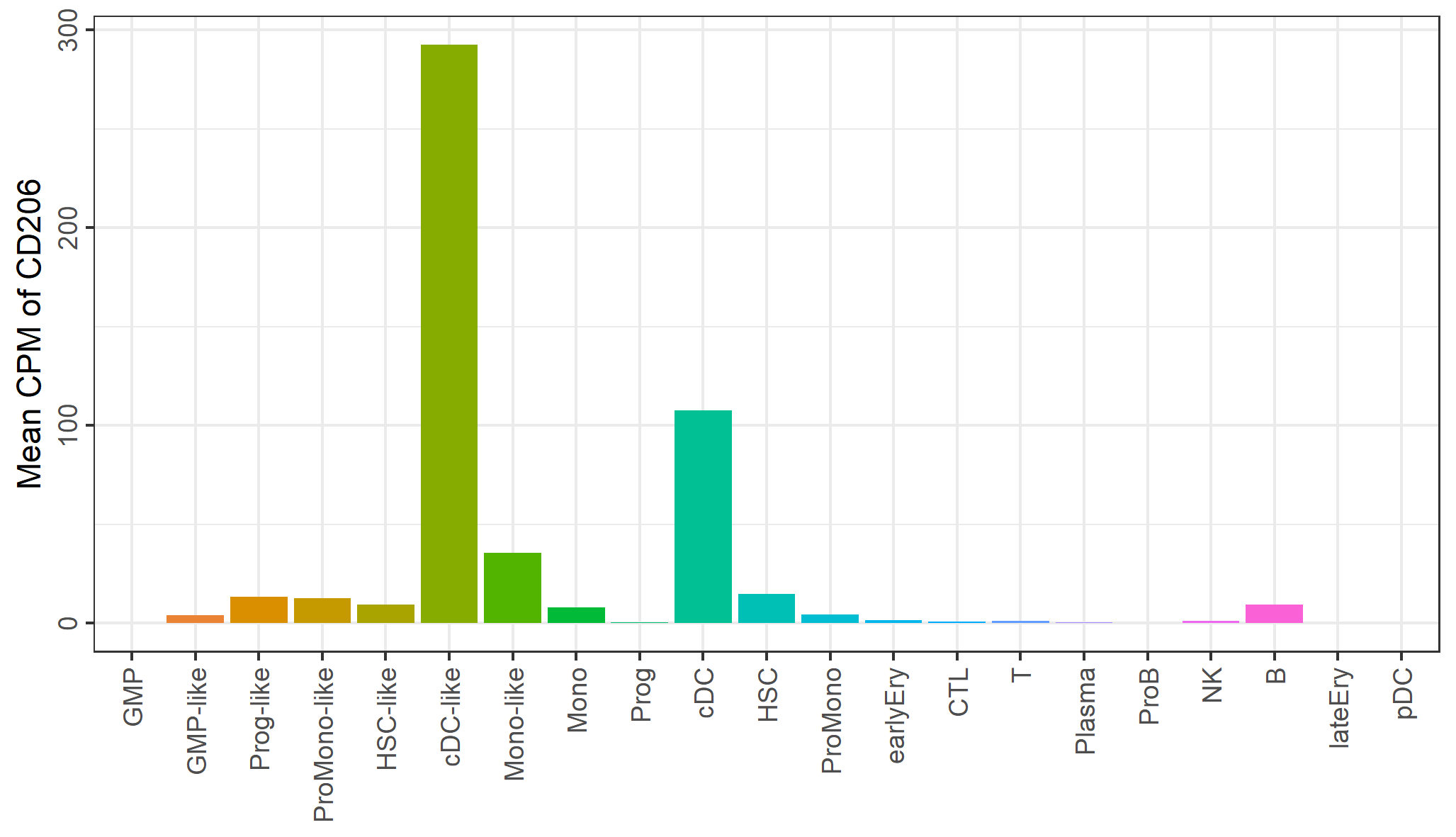

Supplement: Supplementary file 8 [file Image7.TIFF]
